# Days alive and out of hospital after video-assisted thoracoscopic surgery wedge resection in the era of enhanced recovery

**DOI:** 10.1093/bjsopen/zrad144

**Published:** 2023-12-18

**Authors:** Lin Huang, Mikkel Nicklas Frandsen, Henrik Kehlet, René Horsleben Petersen

**Affiliations:** Department of Cardiothoracic Surgery, Copenhagen University Hospital, Rigshospitalet, Copenhagen, Denmark; Section for Surgical Pathophysiology, Copenhagen University Hospital, Rigshospitalet, Copenhagen, Denmark; Section for Surgical Pathophysiology, Copenhagen University Hospital, Rigshospitalet, Copenhagen, Denmark; Department of Cardiothoracic Surgery, Copenhagen University Hospital, Rigshospitalet, Copenhagen, Denmark

## Abstract

**Background:**

Days alive and out of hospital is proposed as a valid and patient-centred quality measure for perioperative care. However, no procedure-specific data exist after pulmonary wedge resection. The aim of this study was to assess the first 90 days alive and out of hospital after video-assisted thoracoscopic surgery wedge resection in an optimized enhanced recovery programme.

**Methods:**

A retrospective analysis of prospectively collected data of consecutive patients undergoing enhanced recovery thoracoscopic wedge resections from January 2021 to June 2022 in a high-volume centre was carried out. All factors leading to hospitalization, readmission, and death were evaluated individually. A logistic regression model was used to evaluate predictors. Additionally, a sensitivity analysis was performed.

**Results:**

A total of 433 patients were included (21.7% (*n* = 94) with non-small cell lung cancer, 47.6% (*n* = 206) with metastasis, 26.8% (*n* = 116) with benign nodules, and 3.9% (*n* = 17) with other lung cancers). The median duration of hospital stay was 1 day. The median of postoperative 30 and 90 days alive and out of hospital was 28 and 88 days respectively. Air leak (112 patients) and pain (96 patients) were the most frequent reasons for reduced days alive and out of hospital from postoperative day 1 to 30, whereas treatment of the original cancer or metastasis (36 patients) was the most frequent reason for reduced days alive and out of hospital from postoperative day 31 to 90. Male sex, reduced lung function, longer dimension of resection margin, pleural adhesions, and non-small cell lung cancer were independent risks, confirmed by a sensitivity analysis.

**Conclusion:**

Days alive and out of hospital within 90 days after enhanced recovery thoracoscopic wedge resection was only reduced by a median of 2 days, mainly due to air leak and pain.

## Teaser

The 90 days alive and out of hospital after enhanced recovery thoracoscopic wedge resection was a median of 88 days. Air leak and pain were the most common reasons for the reduction.

## Introduction

More than 300 million people worldwide undergo surgical procedures each year^1^. Since its introduction in 1995, fast-track surgery, also known as enhanced recovery after surgery (ERAS), has been pivotal in transforming perioperative care^2^. In 2010, the ERAS Society started standardizing procedure-specific perioperative care recommendations in a series of surgeries, including lung surgery^3,4^. Despite the utilization of enhanced recovery after video-assisted thoracoscopic surgery (ERAS VATS), some patients experience delayed discharge after pulmonary lobectomy^5^.

In wedge resection, less pulmonary tissue is removed compared with lobectomy and its use is more frequent than lobectomy according to the databases of the Society of Thoracic Surgeons and the European Society of Thoracic Surgeons^6^. However, no procedure-specific data related to outcomes after wedge resection are available, especially within an ERAS VATS programme.

Days alive and out of hospital (DAOH) is a valid, readily obtained, and patient-centred outcome for quality evaluation in surgical audit and perioperative clinical trials^7,8^. As per current consensus recommendations^9,10^, DAOH serves as a comprehensive metric, reflecting all burdens arising from hospitalizations due to any cause during the specified observation interval. This assessment is based on the maximized status of rehabilitation after surgery without complications. Recently, DAOH has been introduced to quality improvement in different procedure-specific ERAS programmes^11–14^, including ERAS VATS lobectomy^15^.

Therefore, the aim of this study was to assess DAOH after VATS wedge resection following an optimized ERAS programme up to postoperative day (POD) 90 and to identify reasons for reduced DAOH.

## Methods

The Department of Cardiothoracic Surgery at Rigshospitalet has one of the largest volumes of VATS procedures across European institutions. A standardized three-port anterior approach is used for VATS wedge resection^16^. Standard treatment for non-small cell lung cancer (NSCLC) is an anatomical resection (lobectomy or segmentectomy) with systematic lymph node dissection. The indication for a wedge resection in NSCLC is only for patients with a very compromised lung function or co-morbidity that is considered unsuitable for an anatomical resection by multidisciplinary tumour conference. It is recommended to perform lymph node dissection; however, this is up to the surgeon’s clinical judgment. If no preoperative biopsy is obtained, a diagnostic wedge is performed. If benign, no further resection is done; however, if malignant, a completion anatomical resection is performed either during the same procedure or later, depending on the pathology. For metastasis, a wedge resection is standard, unless the location is central or there is multiple metastasis in the same lobe or segment. Sealant or glue is rarely used intraoperatively.

Patients are transferred to a regular ward after 2 h of observation in a post-anaesthesia care unit. As for perioperative care, besides adhering to ERAS guidelines^3^, optimized care includes an intercostal catheter with 0.25% bupivacaine 6 ml/h until the day of chest drain removal and a surgeon-administered paravertebral block (20 ml 0.5% bupivacaine) combined with multimodal opioid-sparing analgesia (paracetamol, ibuprofen, and gabapentin), mobilization on the day of surgery, only one chest drain (Ch 20F, 1F corresponding to one-third millimetre) with −2 cm H_2_O suction on a digital drainage system (Thopaz+, Medela AG, Baar, Switzerland), early removal of the chest drain (air leak less than 20 ml/h for greater than 12 h without a fixed upper limit for serous output), and daily rounds by the operating surgeon^5,17,18^. Discharge criteria are self-mobilization, all lines and the chest drain removed, and no need for inpatient treatment. An X-ray is performed 2 h after removal of the chest drain and post-discharge management after VATS wedge resection is similar to that after VATS lobectomy^19^.

This study was a retrospective analysis of prospectively collected consecutive ERAS thoracoscopic wedge resection data from January 2021 to June 2022 at Rigshospitalet. No formal power calculation for sample size was done. The national electronic medical record system (E-journal) contains exhaustive patient enrolment information necessary to fulfill economic reimbursement prerequisites. Access to the E-journal was facilitated by digital healthcare software (Epic, Madison, WI, USA), through which the authors retrieved the data. Subsequently, the Research Electronic Data Capture (REDCap) tool (Vanderbilt University, Nashville, TN, USA) was utilized to manage and store the collected information.

Inclusion criteria included: age greater than or equal to 18 years; scheduled to undergo VATS wedge resection; residing in East Denmark; and the first operation within this cross-sectional interval. Exclusion criteria included: conversion to lobectomy, segmentectomy, biopsy, or thoracotomy; and incomplete postoperative 90-day follow-up.

Demographics included age, sex, BMI, smoking status, alcohol abuse (greater than 10 units of alcohol/week according to the Danish Health Authority), prior major surgery, and prior oncological therapy. Preoperative clinical characteristics included pulmonary function, ASA grade, age-adjusted Charlson co-morbidity index, and average of long and short axis of the pulmonary nodule measured on CT (in patients with multiple nodules, total values were recorded). Intraoperative characteristics included surgical duration, pleural adhesion status, number of wedges resected, and distribution of wedge resections. Pathological characteristics included diameter of pulmonary nodule (in some patients with multiple nodules, total values were recorded), maximum dimension of resection margin (in patients with multiple nodules, total values were recorded), distance to the visceral pleura (in patients with multiple nodules, the average value was recorded), margin distance (in some patients with multiple nodules, the average value was recorded), pathological diagnosis, lymph node sampling, and TNM stage for NSCLC. Postoperative outcomes included duration of hospital stay, reoperation, ICU admission, emergency room visits, and readmission.

The primary endpoint was DAOH up to POD 90 (DAOH 90) [=90 − (index duration of hospital stay + readmitted duration of hospital stay within postoperative 90 days + the days between the day of death and POD 90)]. Duration of hospital stay and duration of chest drainage were counted as number of nights hospitalized/with chest drain after surgery. No patient was discharged with a chest drain. Secondary endpoints were DAOH 30, reasons for DAOH (stratified by POD 1–30 and 31–90), and predictors of reduced DAOH 90.

All factors associated with hospitalization, readmission, and death were assessed individually as reasons. Multiple reasons for reduced DAOH were recorded. Unplanned surgical/disease-related causes were identified following the ICD (10th revision). Pain was recorded based upon the need for a parenteral opioid after noon on POD 1 in hospital, staying in hospital, or readmission. An air leak was defined as a chest drain that remained in place after noon on POD 1 and pneumothorax/subcutaneous emphysema with need for hospitalization or readmission after chest drain removal. Cardiac dysfunction included postoperative arrhythmia, heart failure, and myocardial infarction. Urinary/renal dysfunction included postoperative urinary tract infection, renal failure, and other urinary and renal disorders. Gastrointestinal dysfunction included postoperative gastrointestinal bleeding, constipation, diarrhoea, nausea, and vomiting. Postoperative bleeding was recorded if a patient required medication (tranexamic acid), a transfusion, chest drain insertion, or reoperation after the index surgery. Wound complications included wound infection, dehiscence, and haematomas. Treatment of the original cancer or metastasis included all relevant factors (for example, adjuvant chemotherapy for primary lung cancer, treatment for brain metastasis (as same histology as lung cancer/metastasis), treatment for rectal cancer (resulting in lung metastasis), and complications or side effects after these subsequent treatments). Treatment of non-surgical or oncological-based disease included all relevant factors not related to surgery or cancer.

Demographics and clinicopathological characteristics were evaluated for predictors of ‘low’ DAOH 90 (defined as values lower than the median of DAOH 90). Continuous variables were transferred into either increase or decrease (for example, surgical duration per 10 min increased and forced expiratory volume in 1 s (FEV_1_) per 0.1 l decreased).

Additionally, sensitivity analyses for identifying predictors were performed using the percentage of DAOH 90 (PDAOH 90) (for example, if a patient has an 80-day DAOH 90, PDAOH 90 is equal to 89% (80 of 90)).

All continuous variables are presented as median (interquartile range (i.q.r.)), because they were non-normally distributed according to Kolmogorov–Smirnov and Shapiro–Wilk tests. All categorical variables are presented as frequency (%). A multiple imputation method was used to impute missing values. The 20 data sets were generated via the chained equation and then each imputed data set was analysed using a logistic regression model and Rubin’s rules to pool results. A logistic regression model using a backwards stepwise method was used to evaluate predictors of ‘low’ DAOH 90 and in the sensitivity analyses. Characteristics with *P* < 0.200 in univariable analysis were used in multivariable analysis for identifying final predictors. The Mann–Whitney *U* test was applied in subgroup analyses. Correlations of DAOH 90 to duration of hospital stay, DAOH 30, readmission, and death, as well as correlations of DAOH for multiple reasons, were tested using the Pearson or Spearman correlation coefficient. A two-sided *P* < 0.050 was considered significant. R Software (version 4.3.2; R Foundation for Statistical Computing, Vienna, Austria) was used to complete all analyses and visualization.

The protocol was approved by the Danish Patient Safety Authority (R-22040550) and the Danish Data Protection Agency (P-2022-464) before the start of the study. The need for informed consent was waived according to the Danish Committee System on Health Research Ethics. STROBE guidelines were used^20^.

## Results

**Fig. 1:**
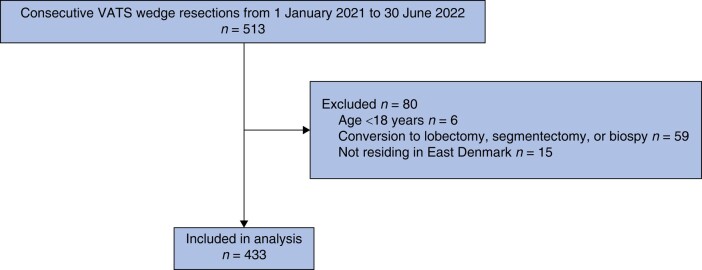
Patient enrolment VATS, video-assisted thoracoscopic surgery.

There were 513 potentially eligible patients and, after exclusions, a total of 433 patients were included in the final analysis. See *Fig. 1*. All patients had an R0 resection. Demographics and preoperative and intraoperative variables are shown in *Table 1* and pathological variables are shown in *Table 2*. Of the patients, 26.8% (*n* = 116), 21.7% (*n* = 94), 47.6% (*n* = 206), and 3.9% (*n* = 17) had benign nodules, NSCLC, metastasis, and other lung cancers (mixed NSCLC and metastasis, small cell lung cancer, B cell lymphadenoma, and pulmonary cancer of unknown primary or metastasis) respectively. The TNM stage for NSCLC is presented in *Table S1*.

**Table 1:** Demographics and preoperative and intraoperative variables (*n* = 433)

Variables	Values
Age (years), median (i.q.r.)	67 (59–75)
**Sex**	
Male	227 (52.4)
Female	206 (47.6)
BMI (kg/m^2^), median (i.q.r.)	25.2 (22.2–28.7)
**Smoking status**	
Never	127 (29.3)
Former smoker	214 (49.4)
Current smoker	92 (21.2)
Alcohol abuse	115 (26.6)
Prior major surgery	206 (47.6)
Prior oncological therapy	166 (38.3)
**Preoperative pulmonary function, median (i.q.r.)**	
FEV_1_ (l)	2.60 (1.98–3.18)
FVC (l)	3.59 (2.82–4.26)
FEV_1_/FVC (%)	74 (68–80)
Percentage of predicted FEV_1_ value	94 (79–109)
Percentage of predicted diffusing capacity for carbon monoxide	71 (59–83)
**ASA grade**	
I–II	92 (21.2)
III	316 (73.0)
IV	25 (5.8)
Age-adjusted Charlson co-morbidity index, median (i.q.r.)	6 (4–8)
Surgical duration (min), median (i.q.r.)	39 (28–53)
Pleural adhesions	71 (16.4)
**Number of wedges resected**	
1	347 (80.1)
≥2	86 (19.9)
**Distribution of wedge resections**	
Left	185 (42.7)
Right	247 (57.0)
Both	1 (0.2)
Average of long and short axis of pulmonary nodules in radiology (mm), median (i.q.r.)	12 (8–17)

Values are *n* (%) unless otherwise indicated. i.q.r., interquartile range; FEV_1_, forced expiratory volume in 1 s; FVC, forced vital capacity.

**Table 2:** Pathological variables (*n* = 433)

Variables	Values
Diameter of pulmonary nodule (mm), median (i.q.r.)	14 (9–20)
Maximum dimension of resection margin (mm), median (i.q.r.)	72 (56–95)
Distance to the visceral pleura (mm), median (i.q.r.)	1 (1–2)
Margin distance (mm), median (i.q.r.)	4 (3–7)
**Pathological diagnosis**	
Benign	116 (26.8)
Non-small cell lung cancer	94 (21.7)
Adenocarcinoma *in situ*/minimally invasive adenocarcinoma	2 (0.5)
Invasive adenocarcinoma	65 (15.0)
Squamous cell carcinoma	16 (3.7)
Adenosquamous carcinoma	2 (0.5)
Carcinoid tumour	4 (0.9)
Large cell carcinoma	5 (1.2)
Metastasis	206 (47.6)
Other lung cancers	17 (3.9)
Mixed non-small cell lung cancer and metastasis	2 (0.5)
B cell lymphadenoma	1 (0.2)
Small cell lung cancer	3 (0.7)
Pulmonary cancer of unknown primary or metastasis	11 (2.5)
**Lymph node sampling**	
No resection	395 (91.2)
Negative	34 (87.9)
Positive	4 (0.9)

Values are *n* (%) unless otherwise indicated. i.q.r., interquartile range.

The median duration of hospital stay was 1 (i.q.r. 1–2) day (*Table 3*). Just one patient was transferred to the ICU during the index hospitalization (due to severe pneumonia leading to dyspnoea and cardiac arrest) and an additional three patients were transferred to the ICU after POD 30 (one due to severe complications after resection of primary cancer, one due to side effects after immunotherapy for primary cancer, and one due to severe complications after treatment of non-surgical or oncological-based disease). A total of five patients experienced reoperation during the index hospitalization (two due to prolonged air leak and three due to postoperative bleeding) and one after POD 30 (due to wound infection). A total of two deaths happened during the index hospitalization (due to severe pneumonia leading to respiratory failure on POD 11 and myocardial infarction leading to heart failure on POD 2); other deaths (4) occurred after POD 30 (one due to severe side effects after immunotherapy leading to respiratory failure and cardiac arrest, one due to oncological-related anaemia leading to serious weakness and malnutrition, one due to suicide, and one due to cancer recurrence with extensive metastasis). Of the 34 patients with emergency room visits up to POD 90, only five patients had visits that were not due to surgical/disease-related reasons. There were 118 patients with readmission during postoperative 90 days (32 patients with readmission related to surgical/disease-related factors).

**Table 3:** Outcomes of postoperative 90 days (*n* = 433)

Outcomes	Values
Duration of hospital stay (days), median (i.q.r.)	1 (1–2)
ICU admission	4 (0.9)
Reoperation	6 (1.4)
Surgical/disease-related emergency room visits	29 (6.7)
Unplanned surgical/disease-related readmission	32 (7.4)
Deaths	6 (1.4)

Values are *n* (%) unless otherwise indicated. i.q.r., interquartile range.

**Fig. 2:**
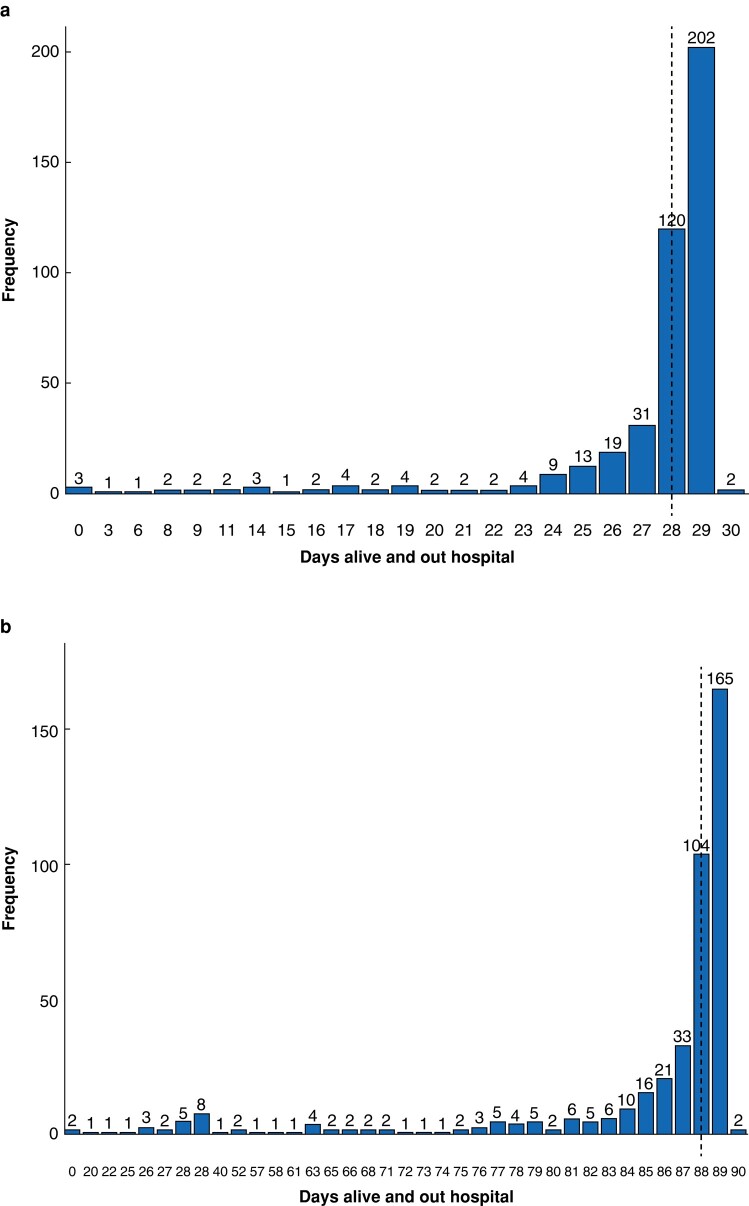
Distribution of days alive and out of hospital (DAOH) after enhanced recovery thoracoscopic wedge resection **a** Thirty days alive and out of hospital. **b** Ninety days alive and out of hospital. Dashed line, median DAOH 30 in (a) and median DAOH 90 in (b).

The median DAOH 30 and DAOH 90 were 28 (i.q.r. 27–29) and 88 (i.q.r. 86–89) days respectively (*Fig. 2*). The median DAOH 30 was 28 (i.q.r. 28–29), 28 (i.q.r. 27–29), 29 (i.q.r. 28–29), and 28 (i.q.r. 26–29) days for benign, NSCLC, metastasis, and other lung cancers respectively, whereas the median DAOH 90 was 88 (i.q.r. 87–89), 87 (i.q.r. 81–88), 88 (i.q.r. 86–89), and 88 (i.q.r. 83–89) days respectively.

**Fig. 3:**
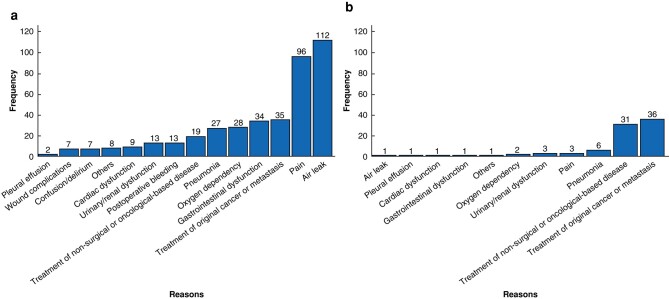
Reasons for reduced days alive and out of hospital **a** Reasons for reduced days alive and out of hospital from postoperative day 1 to 30. Others corresponds to one instance of an allergic reaction with a red rash, one instance of urticaria, three instances of atelectasis, one instance of empyema, one instance of respiratory failure, and one instance of haemopneumothorax. **b** Reasons for reduced days alive and out of hospital from postoperative day 31 to 90. Others corresponds to one instance of pulmonary embolism.

The dominant reasons for reduced DAOH from POD 1 to 30 were air leak and pain in 25.9% (112 of 433) and 22.2% (96 of 433) of patients respectively (*Fig. 3*). Treatment of the original cancer or metastasis (8.3%, 36 of 433) was the most frequent reason for reduced DAOH from POD 31 to 90, followed by treatment of non-surgical or oncological-based disease (7.2%, 31 of 433) (*Fig. 3*).

These various reasons had differing impacts on the reduction in median DAOH 30 and DAOH 90. For instance, patients with air leak had a median DAOH 30 of 27 (i.q.r. 23–28) days and a median DAOH 90 of 87 (i.q.r. 79–88) days. Similarly, patients experiencing pain had a median DAOH 30 of 27 (i.q.r. 24–28) days and a median DAOH 90 of 87 (i.q.r. 81–88) days. In contrast, patients with pneumonia exhibited a median DAOH 30 of 21 (i.q.r. 11–26) days and a median DAOH 90 of 78 (i.q.r. 67–85) days, whereas those undergoing treatment of the original cancer or metastasis had a median DAOH 30 of 28 (i.q.r. 26–29) days and a median DAOH 90 of 84 (i.q.r. 76–87) days (*Table S2*). The correlation coefficients revealed a moderate association between air leak and DAOH 30 (*r*_Spearman_ = −0.540), as well as between treatment of the original cancer or metastasis and DAOH 90 (*r*_Spearman_ = −0.440). With the exception of a moderate association between pneumonia and oxygen dependency, the correlations between other factors remained weak. See *Fig. S1*.

Univariable analysis for ‘low’ DAOH 90 is shown in *Table S3*. Multivariable regression analysis demonstrated that male sex (OR 1.98, 95% c.i. 1.24 to 3.17; *P* = 0.004), FEV_1_ (per 0.1 l decreased; OR 1.99, 95% c.i. 1.78 to 2.01; *P* = 0.003), pleural adhesions (OR 1.88, 95% c.i. 1.10 to 3.24; *P* = 0.022), maximum dimension of resection margin (per 1 mm increased; OR 1.01, 95% c.i. 1.00 to 1.01; *P* = 0.019), and NSCLC (OR 2.45, 95% c.i. 1.36 to 4.43; *P* = 0.003) were predictors of ‘low’ DAOH 90 (*Table 4*).

**Table 4:** Predictors of ‘low’ 90 days alive and out of hospital (<88 days)

Predictors	Multivariable analysis	
	OR (95% c.i.)	*P*
Male sex (reference: female sex)	1.98 (1.24,3.17)	0.004
FEV_1_, per 0.1 l decreased	1.99 (1.78,2.01)	0.003
Pleural adhesions (reference: no)	1.88 (1.10,3.24)	0.022
Maximum dimension of resection margin, per 1 mm increased	1.01 (1.00,1.01)	0.019
**Pathological diagnosis (reference: benign)**		
Non-small cell lung cancer	2.45 (1.36,4.43)	0.003
Metastasis	1.27 (0.76,2.12)	0.355
Others*	1.00 (0.33,3.02)	0.996

*Others corresponds to mix of non-small cell lung cancer and metastasis, small cell lung cancer, B cell lymphadenoma, and pulmonary cancer of unknown primary or metastasis. FEV_1_, forced expiratory volume in 1 s.

Regarding the sensitivity analysis, the results of the univariable analysis are shown in *Table S3*. Male sex (OR 1.89, 95% c.i. 1.18 to 3.04; *P* = 0.009), FEV_1_ (per 0.1 l decreased; OR 1.98, 95% c.i. 1.73 to 2.01; *P* = 0.004), pleural adhesions (OR 1.82, 95% c.i. 1.05 to 3.13; *P* = 0.032), maximum dimension of resection margin (per 1 mm increased; OR 1.01, 95% c.i. 1.00 to 1.02; *P* = 0.019), and NSCLC (OR 2.46, 95% c.i. 1.36 to 4.45; *P* = 0.003) were factors associated with ‘low’ PDAOH 90 (median 98%, i.q.r. 95%–99%) (*Table 5*).

**Table 5:** Sensitivity analysis for predictors of ‘low’ % of 90 days alive and out of hospital (<98%)

Predictors	Multivariable analysis	
	OR (95% c.i.)	*P*
Male sex (reference: female sex)	1.89 (1.18,3.04)	0.009
FEV_1_, per 0.1 l decreased	1.98 (1.73,2.01)	0.004
Pleural adhesions (reference: no)	1.82 (1.05,3.13)	0.032
Maximum dimension of resection margin, per 1 mm increased	1.01 (1.00,1.02)	0.019
**Pathological diagnosis (reference: benign)**		
Non-small cell lung cancer	2.46 (1.36,4.45)	0.003
Metastasis	1.27 (0.76,2.12)	0.357
Others*	1.06 (0.35,3.22)	0.916

*Others corresponds to mix of non-small cell lung cancer and metastasis, small cell lung cancer, B cell lymphadenoma, and pulmonary cancer of unknown primary or metastasis. FEV_1_, forced expiratory volume in 1 s.

Duration of hospital stay (*r*_Pearson_ = −0.214), death (*r*_Spearman_ = −0.197), and DAOH 30 (*r*_Pearson_ = 0.391) had a low association with DAOH 90, whereas readmission (*r*_Spearman_ = −0.592) was moderately associated.

Percentages of missing data were as follows: FEV_1_, 1.4%; percentage of predicted FEV_1_ value, 1.2%; forced vital capacity (FVC), 1.4%; FEV_1_/FVC, 1.4%; percentage of predicted diffusion capacity for carbon monoxide, 3.9%; maximum dimension of resection margin, 1.2%; diameter of pulmonary nodule, 6.1%; distance to the visceral pleura, 1.0%; and margin distance, 1.2%.

## Discussion

With the implementation of VATS, significant benefits in outcomes have been achieved, when compared with open lung surgery^21,22^. However, there are limited procedure-specific data for VATS wedge resection following ERAS programmes such as presented in the present study.

The median duration of hospital stay of 1 day is substantially lower than previous findings (3–6 days) across the European Union, the USA, and China^6,23^, thereby achieving a median DAOH 90 of 88 days. Although the possibility of day surgery in lung resection for selected patients has been reported^24^, further selection criteria and safety data are needed. The present findings offer a basic rationale for further shortening duration of hospital stay without reducing DAOH with day surgery for a selected group of patients.

Importantly, air leak was the main reason for reducing DAOH, as also shown after ERAS VATS lobectomy^15^. Furthermore, air leak was the main reason for prolonged hospitalization (27.7%) and for readmission (18.3%) following an established VATS lobectomy ERAS programme^5,19^. Thus, prevention of postoperative air leak is vital. Prevention includes applying a fissureless technique^25^, the use of tissue sealants^26^, high-quality endoscopic staplers^27^, and minimal suction with a digital chest drainage system^18^. Once a postoperative air leak is identified, it should be addressed in a timely manner to minimize its duration. If the air leak is substantial, a reoperation by VATS is considered. Otherwise, application of an autologous blood patch is an easy, effective, and cheap approach to reduce prolonged air leak^28^. Although models for predicting postoperative air leak^29^ have been published, their implementation and value may be questionable in the real world for clinicians.

The secondary finding was that postoperative pain is the second most important reason for decreasing DAOH. Optimization of pain management in ERAS VATS wedge resection is needed, as pain significantly hinders the return to preoperative functional status^30^. Prolonged chest drain placement results in postoperative pain, pneumonia, empyema, and other adverse events. Therefore, early chest drain removal is recommended after thoracic surgery^31^. Omitting the chest drain after VATS wedge resection (removing the chest drain intraoperatively) is a safe and feasible method to decrease pain for selected patients with negative intraoperative air leak tests^32,33^. However, the general evidence for the effectiveness of this technique is limited. Future studies should study outcomes based on large samples, should be multicentred, and should be RCTs to confirm the safety and benefits. Moreover, a possible strategy to address postoperative pain could be high-dose glucocorticoid administration, as shown in other surgical series^34^.

Furthermore, pneumonia, oxygen dependency, and other postoperative complications were among the factors associated with reduced DAOH. It is conceivable that further optimization of the current perioperative care programme, with a specific emphasis on early chest drain removal, better pain management, and early mobilization^35^, may contribute to improved outcomes. Certainly, improvement of cancer therapy is also essential, with regard to the treatment of the original cancer or metastasis being one of the top three reasons for reduced DAOH, irrespective of overall, early, or late interval of postoperative 90 days.

Male patients, patients with reduced lung function, patients with pleural adhesions, patients with longer dimension of resection margin, and patients with NSCLC had a higher probability of a ‘low’ DAOH 90 in the present study. In the era of advocating individualized therapy, more focus should be on these high-risk patients. Interestingly, age and intraoperative variables were not independent factors reducing DAOH in this cohort.

A strength of the present study is that it is the first detailed assessment of procedure-specific outcomes in unselected ERAS VATS wedge resections using DAOH as a measurement. Also, due to the national register system in East Denmark, the follow-up is nearly 100%. However, the present study has several limitations: the sample size; some missing data; and no formal power calculation of the sample size, possibly leading to the study being underpowered to demonstrate potential impacts on reduced DAOH. Moreover, the cohort consists of patients with both benign and malignant lesions, introducing potential confounders when assessing factors related to oncological treatment for reduced DAOH. Also, the present study is a single-centre study focusing on a hospital with a high volume of VATS and a specialized ERAS programme, which may limit the generalization of the results. Finally, the retrospective study design may limit the findings, despite the complete follow-up.

DAOH within the first 90 days after VATS wedge resection in an ERAS setting was only reduced by a median of 2 days. The main reasons for reduced DAOH were air leak and pain. DAOH assessment may provide a patient-centred parameter to guide future optimization of treatment.

## Supplementary Material

zrad144_Supplementary_Data

## Data Availability

Data available on request from the authors.

## References

[1] Meara JG, Leather AJ, Hagander L, Alkire BC, Alonso N, Ameh EA et al Global surgery 2030: evidence and solutions for achieving health, welfare, and economic development. Lancet 2015;386:569–624 25924834 10.1016/S0140-6736(15)60160-X

[2] Kehlet H . Multimodal approach to control postoperative pathophysiology and rehabilitation. Br J Anaesth 1997;78:606–617 9175983 10.1093/bja/78.5.606

[3] Batchelor TJP, Rasburn NJ, Abdelnour-Berchtold E, Brunelli A, Cerfolio RJ, Gonzalez M et al Guidelines for enhanced recovery after lung surgery: recommendations of the Enhanced Recovery After Surgery (ERAS^®^) Society and the European Society of Thoracic Surgeons (ESTS). Eur J Cardiothorac Surg 2019;55:91–115 30304509 10.1093/ejcts/ezy301

[4] Kehlet H . Enhanced postoperative recovery: good from afar, but far from good? Anaesthesia 2020;75:e54–e61 31903577 10.1111/anae.14860

[5] Huang L, Kehlet H, Petersen RH. Reasons for staying in hospital after video-assisted thoracoscopic surgery lobectomy. BJS Open 2022;6:zrac050 10.1093/bjsopen/zrac050 PMC9070644 35511502

[6] Seder CW, Salati M, Kozower BD, Wright CD, Falcoz PE, Brunelli A et al Variation in pulmonary resection practices between the Society of Thoracic Surgeons and the European Society of Thoracic Surgeons general thoracic surgery databases. Ann Thorac Surg 2016;101:2077–2084 27021033 10.1016/j.athoracsur.2015.12.073

[7] Myles PS, Shulman MA, Heritier S, Wallace S, McIlroy DR, McCluskey S et al Validation of days at home as an outcome measure after surgery: a prospective cohort study in Australia. BMJ Open 2017;7:e015828 10.1136/bmjopen-2017-015828 PMC5629653 28821518

[8] Bell M, Eriksson LI, Svensson T, Hallqvist L, Granath F, Reilly J et al Days at home after surgery: an integrated and efficient outcome measure for clinical trials and quality assurance. EClinicalMedicine 2019;11:18–26 31317130 10.1016/j.eclinm.2019.04.011 PMC6610780

[9] Buggy DJ, Freeman J, Johnson MZ, Leslie K, Riedel B, Sessler DI et al Systematic review and consensus definitions for standardised endpoints in perioperative medicine: postoperative cancer outcomes. Br J Anaesth 2018;121:38–44 29935592 10.1016/j.bja.2018.03.020

[10] Domenghino A, Walbert C, Birrer DL, Puhan MA, Clavien PA. Consensus recommendations on how to assess the quality of surgical interventions. Nat Med 2023;29:811–822 37069361 10.1038/s41591-023-02237-3

[11] Jorgensen CC, Petersen PB, Kehlet H. Days alive and out of hospital after fast-track total hip and knee arthroplasty: an observational cohort study in 16 137 patients. Br J Anaesth 2019;123:671–678 31474350 10.1016/j.bja.2019.07.022

[12] Maibom SL, Røder MA, Poulsen AM, Thind PO, Salling ML, Salling LN et al Morbidity and days alive and out of hospital within 90 days following radical cystectomy for bladder cancer. Eur Urol Open Sci 2021;28:1–8 34337519 10.1016/j.euros.2021.03.010 PMC8317890

[13] Scott SI, Madsen AKO, Rubek N, Kehlet H, von Buchwald C. Days alive and out of hospital after treatment for oropharyngeal squamous cell carcinoma with primary transoral robotic surgery or radiotherapy—a prospective cohort study. Acta Otolaryngol 2021;141:193–196 33151114 10.1080/00016489.2020.1836395

[14] Larsen MHH, Scott SI, Kehlet H, von Buchwald C. Days alive and out of hospital a validated patient-centred outcome to be used for patients undergoing transoral robotic surgery: protocol and perspectives. Acta Otolaryngol 2021;141:95–98 33107363 10.1080/00016489.2020.1814964

[15] Huang L, Frandsen MN, Kehlet H, Petersen RH. Days alive and out of hospital after enhanced recovery video-assisted thoracoscopic surgery lobectomy. Eur J Cardiothorac Surg 2022;62:ezac148 35234866 10.1093/ejcts/ezac148

[16] Hansen HJ, Petersen RH. Video-assisted thoracoscopic lobectomy using a standardized three-port anterior approach—the Copenhagen experience. Ann Cardiothorac Surg 2012;1:70–76 23977470 10.3978/j.issn.2225-319X.2012.04.15 PMC3741716

[17] Wildgaard K, Petersen RH, Hansen HJ, Moller-Sorensen H, Ringsted TK, Kehlet H. Multimodal analgesic treatment in video-assisted thoracic surgery lobectomy using an intraoperative intercostal catheter. Eur J Cardiothorac Surg 2012;41:1072–1077 22219442 10.1093/ejcts/ezr151

[18] Holbek BL, Christensen M, Hansen HJ, Kehlet H, Petersen RH. The effects of low suction on digital drainage devices after lobectomy using video-assisted thoracoscopic surgery: a randomized controlled trial. Eur J Cardiothorac Surg 2019;55:673–681 30445572 10.1093/ejcts/ezy361

[19] Huang L, Frandsen MN, Kehlet H, Petersen RH. Early and late readmissions after enhanced recovery thoracoscopic lobectomy. Eur J Cardiothorac Surg 2022;62:ezac385 35880263 10.1093/ejcts/ezac385

[20] von Elm E, Altman DG, Egger M, Pocock SJ, Gotzsche PC, Vandenbroucke JP et al Strengthening the Reporting of Observational Studies in Epidemiology (STROBE) statement: guidelines for reporting observational studies. BMJ 2007;335:806–808 17947786 10.1136/bmj.39335.541782.AD PMC2034723

[21] Bendixen M, Jorgensen OD, Kronborg C, Andersen C, Licht PB. Postoperative pain and quality of life after lobectomy via video-assisted thoracoscopic surgery or anterolateral thoracotomy for early stage lung cancer: a randomised controlled trial. Lancet Oncol 2016;17:836–844 27160473 10.1016/S1470-2045(16)00173-X

[22] Lim E, Batchelor TJP, Dunning J, Shackcloth M, Anikin V, Naidu B et al Video-assisted thoracoscopic or open lobectomy in early-stage lung cancer. NEJM Evid 2022;1:EVIDoa2100016 10.1056/EVIDoa2100016 38319202

[23] Qiu B, Yan W, Chen K, Fu X, Hu J, Gao S et al A multi-center evaluation of a powered surgical stapler in video-assisted thoracoscopic lung resection procedures in China. J Thorac Dis 2016;8:1007–1013 27162678 10.21037/jtd.2016.03.88 PMC4842836

[24] Dong Y, Li J, Chang J, Song W, Wang Y, Wang Y et al Video-assisted thoracoscopic day surgery for patients with pulmonary nodules: a single-center clinical experience of 200 cases. Cancer Manag Res 2021;13:6169–6179 34393510 10.2147/CMAR.S324165 PMC8354674

[25] Stamenovic D, Bostanci K, Messerschmidt A, Jahn T, Schneider T. Fissureless fissure-last video-assisted thoracoscopic lobectomy for all lung lobes: a better alternative to decrease the incidence of prolonged air leak? Eur J Cardiothorac Surg 2016;50:118–123 26792925 10.1093/ejcts/ezv455

[26] Moser C, Opitz I, Zhai W, Rousson V, Russi EW, Weder W et al Autologous fibrin sealant reduces the incidence of prolonged air leak and duration of chest tube drainage after lung volume reduction surgery: a prospective randomized blinded study. J Thorac Cardiovasc Surg 2008;136:843–849 18954621 10.1016/j.jtcvs.2008.02.079

[27] Tantraworasin A, Seateang S, Bunchungmongkol N. Staplers versus hand-sewing for pulmonary lobectomy: randomized controlled trial. Asian Cardiovasc Thorac Ann 2014;22:309–314 24585907 10.1177/0218492313491754

[28] Shackcloth MJ, Poullis M, Jackson M, Soorae A, Page RD. Intrapleural instillation of autologous blood in the treatment of prolonged air leak after lobectomy: a prospective randomized controlled trial. Ann Thorac Surg 2006;82:1052–1056 16928534 10.1016/j.athoracsur.2006.04.015

[29] Zaraca F, Pipitone M, Feil B, Perkmann R, Bertolaccini L, Curcio C et al Predicting a prolonged air leak after video-assisted thoracic surgery, is it really possible? Semin Thorac Cardiovasc Surg 2021;33:581–592 32853737 10.1053/j.semtcvs.2020.08.012

[30] Huang L, Kehlet H, Petersen RH. Functional recovery after discharge in enhanced recovery video-assisted thoracoscopic lobectomy: a pilot prospective cohort study. Anaesthesia 2022;77:555–561 35261025 10.1111/anae.15682

[31] Bjerregaard LS, Jensen K, Petersen RH, Hansen HJ. Early chest tube removal after video-assisted thoracic surgery lobectomy with serous fluid production up to 500 ml/day. Eur J Cardiothorac Surg 2014;45:241–246 23872457 10.1093/ejcts/ezt376

[32] Holbek BL, Hansen HJ, Kehlet H, Petersen RH. Thoracoscopic pulmonary wedge resection without post-operative chest drain: an observational study. Gen Thorac Cardiovasc Surg 2016;64:612–617 27510705 10.1007/s11748-016-0692-6

[33] Huang L, Kehlet H, Holbek BL, Jensen TK, Petersen RH. Efficacy and safety of omitting chest drains after video-assisted thoracoscopic surgery: a systematic review and meta-analysis. J Thorac Dis 2021;13:1130–1142 33717586 10.21037/jtd-20-3130 PMC7947539

[34] Nielsen NI, Kehlet H, Gromov K, Troelsen A, Husted H, Varnum C et al High-dose steroids in high pain responders undergoing total knee arthroplasty: a randomised double-blind trial. Br J Anaesth 2022;128:150–158 34749994 10.1016/j.bja.2021.10.001 PMC8787770

[35] Huang L, Kehlet H, Petersen RH. Effect of posture on pulmonary function and oxygenation after fast-tracking video-assisted thoracoscopic surgery (VATS) lobectomy: a prospective pilot study. Perioper Med (Lond) 2021;10:26 34470657 10.1186/s13741-021-00199-z PMC8411530

