# Supplementary material for: Days alive and out of hospital after video-assisted thoracoscopic surgery wedge resection in the era of enhanced recovery
Source: BJS Open. 2023 Dec 18;7(6):zrad144. doi: 10.1093/bjsopen/zrad144 (PMC10726402; doi:10.1093/bjsopen/zrad144)
Supplement: zrad144_Supplementary_Data [file zrad144_supplementary_data.docx]

**Days alive and out of hospital after**

**video-assisted thoracoscopic surgery wedge resection in the era of enhanced recovery**

Lin Huang MD, PhD^a^, Mikkel Nicklas Frandsen MD^b^,

Henrik Kehlet MD, PhD^b^, René Horsleben Petersen MD, PhD^a^

^a^Department of Cardiothoracic Surgery, Copenhagen University Hospital, Rigshospitalet, Copenhagen, Denmark. ^b^Section for Surgical Pathophysiology, Copenhagen University Hospital, Rigshospitalet, Copenhagen, Denmark.

**Corresponding author:**

René Horsleben Petersen, MD, PhD, Department of Cardiothoracic Surgery, Copenhagen University Hospital, Rigshospitalet, Blegdamsvej 9, 2100 Copenhagen Ø, Denmark. Tel: +45 35450525; e-mail: rene.horsleben.petersen@regionh.dk

**Supplementary Materials - Index**

| **Supplementary Figures and Tables** |  |
| --- | --- |
| Table S1 | *page 2* |
| Table S2 | *page 3* |
| Table S3 | *page 4* |
| Figure S1 | *page 5* |

**Table S1. The TNM stage for non-small cell lung cancer (n=94).**

| **TNM stage** | **n (% of patients with non-small cell lung cancer)** |
| --- | --- |
| TisN0M0 | 1 (1.1%) |
| T1aN0M0 | 17 (18.1%) |
| T1bN0M0 | 26 (27.7%) |
| T1cN0M0 | 12 (12.8%) |
| T2aN0M0 | 4 (4.3%) |
| T3N0M0 | 2 (2.1%) |
| T1aN2M0 | 1 (1.1%) |
| T1bN2M0 | 1 (1.1%) |
| T2aN2M0 | 1 (1.1%) |
| T3N2M0 | 1 (1.1%) |
| T1a(mi)NxM0 | 1 (1.1%) |
| T1aNxM0 | 10 (10.6%) |
| T1bNxM0 | 14 (14.9%) |
| T1cNxM0 | 1 (1.1%) |
| T2aNxM0 | 2 (2.1%) |

**Table S2. Days alive and out of hospital of various reasons.**

| **Reasons for reduced DAOH** | **DAOH 30, day, median (IQR)** | **DAOH 90, day, median (IQR)** |
| --- | --- | --- |
| Air leak | 27 (23-28) | 87 (79-88) |
| Pain | 27 (24-28) | 87 (81-88) |
| Treatment to original cancer or metastasis | 28 (26-29) | 84 (76-87) |
| Treatment to non-surgical or oncological-based disease | 27 (25-28) | 82 (57-86) |
| Gastrointestinal dysfunction | 26 (24-28) | 85 (80-88) |
| Oxygen dependency | 25 (15-28) | 85 (72-87) |
| Pneumonia | 21 (11-26) | 78 (67-85) |
| Postoperative bleeding | 27 (16-28) | 86 (70-88) |
| Urinary/renal dysfunction | 26 (13-28) | 82 (73-88) |
| Cardiac dysfunction | 27 (25-29) | 85 (77-87) |
| Confusion/delirium | 19 (3-27) | 79 (63-87) |
| Wound complications | 28 (18-28) | 87 (80-88) |
| Pleural effusion | 19 (5-30) | 78 (55-83) |
| Others | 16 (5-25) | 65 (14-83) |

DAOH: days alive and out of hospital; DAOH 30: 30 days alive and out of hospital; DAOH 90: 90 days alive and out of hospital; IQR: interquartile range.

**Table S3. Univariable analysis for “low” 90 days alive and out of hospital (DAOH 90 < 88 days) and “low” percentage of DAOH 90 (PDAOH 90 < 98%).**

| **Variables** | **Univariable analysis for “low” DAOH 90** | | **Univariable analysis for “low” PDAOH 90** | |
| --- | --- | --- | --- | --- |
|  | **OR (95% CI)** | **P value** | **OR (95% CI)** | **P value** |
| Age, per 10 years increased | 1.18 (1.00 to 1.40) | .052 | 1.14 (0.97 to 1.34) | .110 |
| Male gender (reference: Female gender) | 1.32 (0.90 to 1.96) | .160 | 1.16 (0.57 to 1.24) | .179 |
| BMI, per 5 kg/m^2^ increased | 0.94 (0.78 to 1.13) | .484 | 0.91 (0.76 to 1.09) | .310 |
| Smoking status (reference: Never) |  |  |  |  |
| Former smoker | 1.67 (1.04 to 2.67) | .033 | 1.35 (0.86 to 2.67) | .191 |
| Current smoker | 1.71 (0.97 to 3.01) | .062 | 1.48 (0.97 to 3.01) | .166 |
| Alcohol abuse (reference: No) | 1.17 (0.75 to 1.82) | .497 | 1.20 (0.78 to 1.85) | .415 |
| Prior major surgery (reference: No) | 1.26 (0.86 to 1.87) | .239 | 1.24 (0.84 to 1.83) | .282 |
| Prior oncological therapy (reference: No) | 0.71 (0.47 to 1.07) | .098 | 0.72 (0.49 to 1.07) | .106 |
| FEV_1_, per 0.1 L decreased | 1.30 (1.11 to 1.44) | .003 | 1.25 (1.06 to 1.40) | .011 |
| FVC, per 0.1 L decreased | 1.22 (1.06 to 1.36) | .010 | 1.25 (1.10 to 1.38) | .003 |
| FEV_1_/FVC, per 5% decreased | 1.00 (0.96 to 1.04) | .706 | 1.00 (0.98 to 1.01) | .302 |
| FEV_1_%_pre_, per 5% decreased | 1.00 (1.00 to 1.01) | .013 | 1.00 (0.99 to 1.00) | .131 |
| DLCO%_pre_, per 5% decreased | 1.00 (1.00 to 1.01) | .002 | 1.00 (1.00 to 1.01) | < .001 |
| ASA score (reference: I-II) |  |  |  |  |
| III | 1.10 (0.68 to 1.79) | .694 | 1.08 (0.67 to 1.72) | .763 |
| IV | 2.03 (0.83 to 4.97) | .120 | 3.69 (1.17 to 11.63) | .026 |
| CCI, per 1 score decreased | 1.04 (0.96 to 1.12) | .344 | 1.04 (0.96 to 1.12) | .486 |
| Surgical duration, per 10 min increased | 1.03 (0.97 to 1.08) | .360 | 1.03 (0.95 to 1.11) | .360 |
| Pleural adhesions (reference: No) | 2.06 (1.24 to 3.45) | .006 | 1.74 (1.00 to 3.05) | .051 |
| Number of wedges resected ≥2 (reference: 1) | 1.42 (0.88 to 2.30) | .148 | 1.38 (0.84 to 2.28) | .198 |
| Surgery on the right hemithorax (reference: Left) | 1.15 (0.77 to 1.70) | .498 | 1.10 (0.75 to 1.63) | .620 |
| Average of long and short axis of pulmonary nodules in radiology, per 1 mm increased | 1.04 (1.01 to 1.06) | .004 | 1.03 (1.01 to 1.06) | .016 |
| Diameter of pulmonary nodules, per 1 mm increased | 1.03 (1.01 to 1.05) | .001 | 1.02 (1.00 to 1.04) | .058 |
| Maximum dimension of resection margin, per 1 mm increased | 1.01 (1.00 to 1.01) | .010 | 1.01 (1.00 to 1.01) | .042 |
| Distance to the visceral pleural, per 1 mm decreased | 1.04 (0.95 to 1.14) | .414 | 1.05 (0.95 to 1.16) | .337 |
| Margin distance, per 1mm decreased | 1.02 (0.99 to 1.06) | .220 | 1.01 (0.97 to 1.05) | .735 |
| Pathological diagnosis (reference: Benign) |  |  |  |  |
| Non-small cell lung cancer | 2.87 (1.63 to 5.06) | < .001 | 2.00 (1.09 to 3.67) | .025 |
| Metastasis | 1.07 (0.66 to 1.75) | .783 | 1.26 (0.48 to 1.18) | .209 |
| Others^*^ | 1.21 (0.42 to 3.53) | .724 | 1.32 (0.25 to 1.91) | .473 |
| Lymph node sampling (reference: No resection) |  |  |  |  |
| Negative | 1.36 (0.67 to 2.76) | .393 | 1.83 (0.83 to 4.03) | .312 |
| Positive | 1.72 (0.24 to 12.37) | .588 | 1.98 (0.20 to 19.20) | .556 |

^*^Others in pathological diagnosis included mixed of non-small cell lung cancer and metastasis, small cell lung cancer, B cell lymphadenoma, and cancer without ensured original histology.

ASA: American Society of Anesthesiologists classification; BMI: body mass index; CCI: Charlson Comorbidity index; CI: confidence interval; DLCO% _pre_: percentage of predicted diffusing capacity for carbon monoxide; FEV_1_: forced expiratory volume in 1 s; FEV_1_%_pre_: percentage of predicted FEV_1_ value; FVC: force vital capacity; OR: odds ratio.

**Figure S1. Correlations among days alive and out of hospital after enhanced recovery thoracoscopic wedge resection and various reasons for reduction.**

**
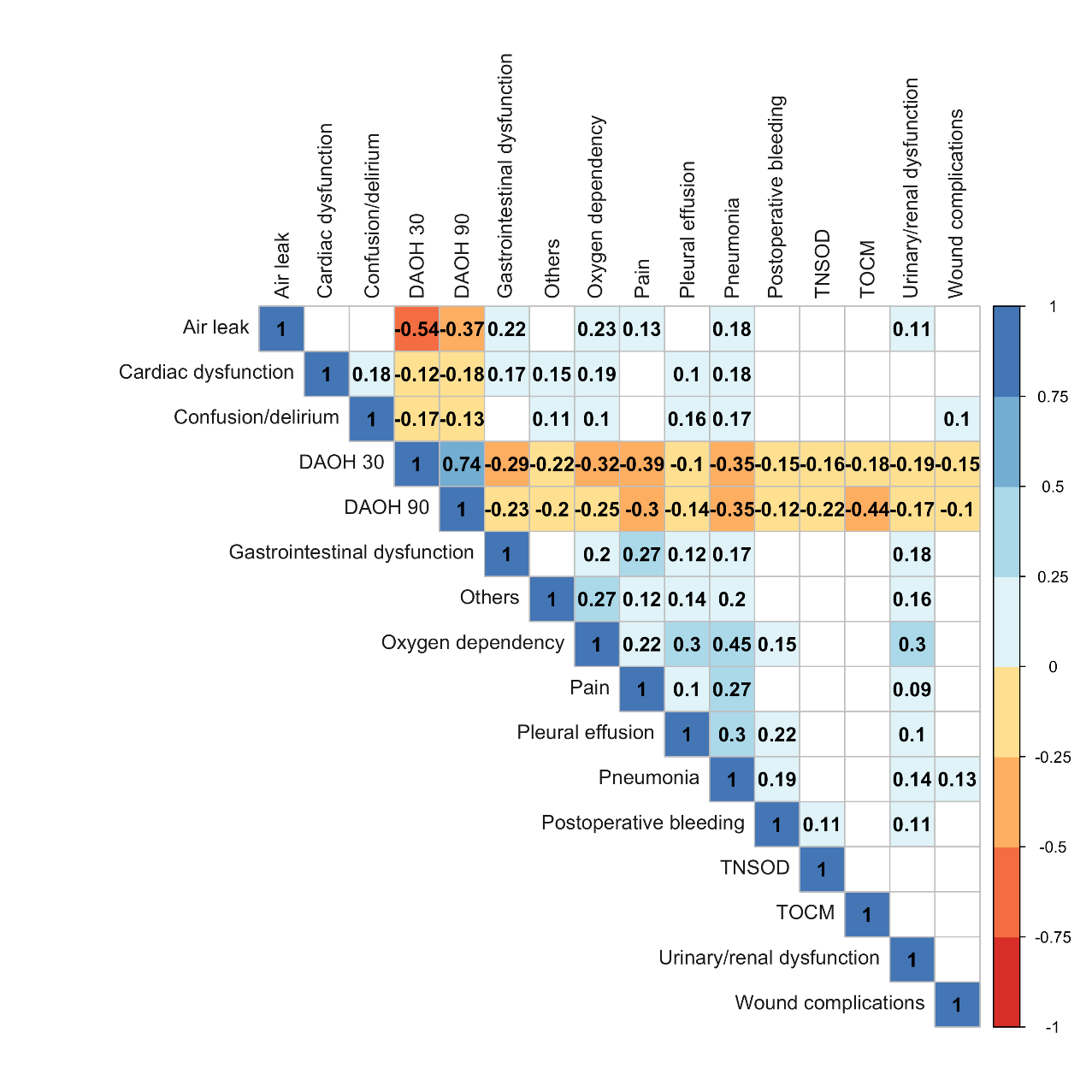
**

The numbers in each square represent correlation coefficients that were analyzed using Spearman's rank correlation. In the representation, a blue color in each square or a positive number indicates a positive correlation between the two factors. As the color darkens or the number approaches 1, the strength of the positive associations increases. Conversely, as the color shifts from yellow to red or the number approaches -1, the strength of the negative associations intensifies. The white squares mean no associations between the two factors.

DAOH 30: 30 days alive and out of hospital; DAOH 90: 90 days alive and out of hospital; TNSOD: treatment to non-surgical or oncological-based disease; TOCM: treatment to original cancer or metastasis.
